# Hybrid System of Magnetic Nanoparticles and Cashew-Gum Nanoparticles Induce Apoptosis and Tumor Necrosis in Colorectal Cancer

**DOI:** 10.1021/acsomega.5c06166

**Published:** 2025-12-05

**Authors:** Vinícius B. Garcia, Cainã O. G. da Silva, Luiz H. S. Gasparotto, Carlos A. M. Iglesias, Isadora L. G. da Silva, Emily L. Oliveira, Raelle F. Gomes, Regina C. M. de Paula, Rosemayre S. Freire, Felipe Bohn, Raimundo F. de Araújo Júnior

**Affiliations:** 1 Inflammation and Cancer Research Laboratory, Department of Morphology, 28123 Federal University of Rio Grande do Norte (UFRN), Natal, Rio Grande do Norte 59078-970, Brazil; 2 Postgraduate Program in Structural and Functional Biology, Department of Morphology, 28123 Federal University of Rio Grande do Norte (UFRN), Natal, Rio Grande do Norte 59.072-970, Brazil; 3 Institute of Chemistry, Federal University of Mato Grosso (UFMT), Cuiabá, Mato Grosso 78060-900, Brazil; 4 Department of Physics, 89113 State University of Rio Grande do Norte, Mossoró, Rio Grande do Norte 59610-090, Brazil; 5 Postgraduate Program in Health Sciences, 28123 Federal University of Rio Grande do Norte (UFRN), Natal, Rio Grande do Norte 59078-970, Brazil; 6 Department of Organic and Inorganic Chemistry, Federal University of Ceará (UFC), Fortaleza, Ceará 60455-760, Brazil; 7 Department of Physics, Federal University of Ceará (UFC), Fortaleza 60455-760, Brazil; 8 Department of Physics, Federal University of Rio Grande do Norte, Natal, Rio Grande do Norte 59078-900, Brazil

## Abstract

Current therapies for colorectal cancer (CRC) are often limited by drug resistance, systemic toxicity, and tumor recurrence. To address these challenges, a multifunctional nanosystem for enhanced drug delivery was developed. The system is composed of cashew gum nanoparticles (CGNPs) coloaded with oxaliplatin (OXA) and retinoic acid (RA), combined with magnetic core–shell nanoparticles (CSNPs). Following synthesis and characterization, *in vitro* assays in CT-26 cells showed efficient nanoparticle internalization and significant dose-dependent viability reduction, with magnetic stimulation increasing late apoptosis. *In vivo* studies using a murine CRC model revealed enhanced tumor necrosis, reduced anaplastic cell frequency, suppressed COX-2 expression, and altered oxidative stress markers, such as SOD and GPX-1. Although tumor volume did not differ significantly between groups, histological and molecular analyses confirmed substantial antitumor effects, particularly with RA-functionalized formulations. The data suggest magnetic stimulation improved nanoparticle uptake, while RA acted as both a cytotoxic agent and a modulator of cellular uptake and inflammation. Overall, the nanosystem promoted apoptosis, tumor necrosis, and biochemical modulation. These findings lay a promising foundation for future research to investigate the nanosystem’s biodistribution, pharmacokinetics, and synergistic effects with immunotherapeutic agents.

## Graphical abstract

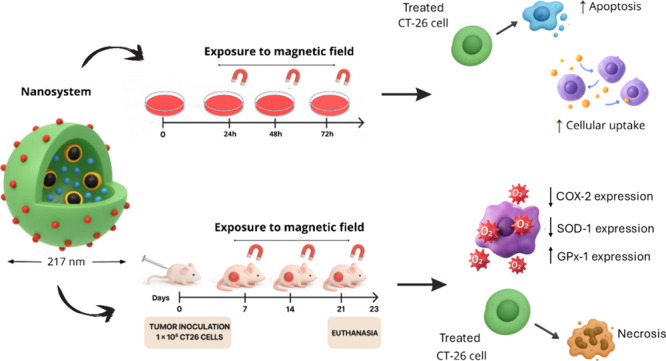

## 1 Introduction

Among the most common and deadly cancers in the world, colorectal cancer (CRC) is the second most common cause of cancer-related deaths and the third most frequently diagnosed cancer type.^1^ Due to aging populations, dietary changes, and restricted access to preventive healthcare, the prevalence of colorectal cancer (CRC) is continuously increasing worldwide, especially in low- and middle-income countries. The prognosis for advanced or metastatic colorectal cancer is still dismal, even with the availability of screening programs and improvements in surgical techniques. This is particularly true for patients who become resistant to current therapies.^1^

At the moment, systemic chemotherapy using drugs like 5-fluorouracil and oxaliplatin is used in conjunction with surgical resection as the primary treatment for colorectal cancer. However, a number of resistance mechanisms, such as drug efflux, enhanced DNA repair, and inhibition of apoptotic pathways, commonly impede its clinical success. Additionally, traditional chemotherapies frequently result in severe off-target toxicity, which lowers treatment efficacy and compromises patients’ quality of life.^1^ These limitations highlight an urgent need for alternative therapeutic strategies capable of selectively targeting tumor cells and modulating the tumor microenvironment.

Given this, nanotechnology presents revolutionary possibilities for enhance cancer care. Nanoparticle-based platforms have the potential to improve drug accumulation at the tumor site, decrease systemic toxicity, and enhance targeted delivery and controlled release of chemotherapeutic agents. A variety of nanomaterials, such as liposomes, dendrimers, and polymeric nanoparticles, have shown promise in preclinical and early clinical studies on colorectal cancer specifically.^2^ These nanosystems can also be functionalized with bioactive compounds to confer additional therapeutic or imaging capabilities.

Retinoids, especially all-trans-retinoic acid (ATRA), are notable among these substances because of their strong antitumor effects. Retinoids mainly affect the expression of genes involved in immune regulation, apoptosis, and differentiation through nuclear receptors (RAR/RXR). Research has demonstrated that by altering macrophage polarization and preventing the epithelial-mesenchymal transition (EMT) in colorectal cancer models, ATRA can inhibit tumor growth and improve sensitivity to immune checkpoint blockade.^3,4^ These findings support the incorporation of retinoic acid into multimodal therapeutic platforms.

Cashew gum (CG), a natural polysaccharide derived from Anacardium occidentale L., is another intriguing ingredient for drug delivery systems. A biodegradable and biocompatible polymer, CG has been extensively studied for use in pharmaceutical applications, especially in nanoparticle formulations.^5^ CG-based nanoparticles are appealing candidates for combinatorial nanomedicine because they have demonstrated selective antitumor activity in vivo without causing systemic toxicity.^6^ Their potential as nanocarriers is further supported by their capacity to stabilize and encapsulate therapeutic agents.

Simultaneously, magnetic nanoparticles have emerged as innovative tools for cancer treatment. Active navigation through biological fluids and tissues is made possible by these magnetically controlled micro- and nanoscale devices, which can move independently or under external guidance. They can produce cytotoxic effects by inducing oxidative stress or magnetic hyperthermia, or they can release therapeutic agents in a controlled and localized manner once they are directed to the tumor site.^7–9^ When integrated into multifunctional platforms, magnetic nanoparticles can significantly enhance the precision and potency of anticancer therapies.

The goal of the current study was to create a multifunctional nanotherapeutic system using cashew gum nanoparticles (CGNP) coloaded with retinoic acid (RA) and oxaliplatin (OXA) in conjunction with magnetic nanoparticles (CSNP), taking into account the individual advantages of these components. The CSNPs in this formulation are designed as core–shell nanostructures, with a gold (Au) shell providing high biocompatibility, structural stability, and surface functionalization potential, and a magnetite (FeO_4_) core that provides magnetic responsiveness and guidance. The nanoparticles’ core–shell design allows them to be magnetically guided toward tumor tissues, while also enhancing cytotoxic mechanisms through oxidative stress modulation and facilitating cellular uptake. The combinatorial method was created to (i) improve drug delivery and tumor targeting through magnetic guidance, (ii) induce more oxidative stress and apoptosis in cancer cells, and (iii) alter the inflammatory milieu to inhibit tumor growth. The need for less harmful and more efficient treatment methods for colorectal cancer has led to the possibility that this integrated nanoplatform could be a viable substitute for, or complement to, existing chemotherapy treatments. Chemotherapy, immunomodulation, and magnetically enhanced delivery systems may be combined in future clinical strategies if it is successfully implemented.

## 2 Results

### 2.1 Characterization of the Nanoparticles

The physicochemical properties of the synthesized formulations were characterized by Dynamic Light Scattering (DLS) and are summarized in Table 1.The cashew gum-based formulations (CGNP, CGNP+OXA, and CGNP+OXA+CSNP+RA) exhibited small hydrodynamic diameters, ranging from 197.0 ± 1.0 nm to 217.0 ± 1.5 nm. The Polydispersity Index (PDI) values for these formulations were low, ranging between 0.03 ± 0.02 and 0.06 ± 0.01, indicating a narrow and homogeneous size distribution.

**Table 1:** Physicochemical Properties of Cashew Gum Nanoparticles (CGNP) and Core-shell Nanoparticles (CSNP), as well as Determination of the Load of Oxaliplatin (OXA) and Retinoic Acid (RA) in Each Nanosystem^a^

Formulations	Size ± SD (nm)	PDI ± SD.	Zeta Potential ± SD (mV)	OXA Loading (mg/mL)	RA Loading (μg/mg)
CGNP	197.0 ± 1.0	0.04 ± 0.02	–37 ± 0.5	-	-
CGNP+OXA	203.0 ± 1.6	0.03 ± 0.02	–43 ± 0.7	3.3	-
CGNP+OXA+CSNP+RA	217.0 ± 1.5	0.06 ± 0.01	–36 ± 0.2	3.3	30.0
CSNP	1699.6 ± 520.6	0.40 ± 0.3	–30 ± 0.1	-	-

a The characterization included the description of the average size, polydispersity index (PDI) and zeta potential, obtained by means of dynamic light scattering (DLS).

In contrast, the isolated Core–Shell Nanoparticles (CSNP) presented a significantly larger measured size of 1699.6 ± 520.6 nm and a higher PDI of 0.40 ± 0.3, suggesting a tendency for aggregation or clustering in aqueous suspension. All formulations displayed a negative surface charge, with Zeta Potential values ranging from −30 ± 0.1 mV for CSNP to −43 ± 0.7 mV for CGNP+OXA. This negative charge plays a crucial role in nanoparticle stability in biological environments. The drug loading analysis confirmed the successful encapsulation of the therapeutic agents. Both the CGNP+OXA and the CGNP+OXA+CSNP+RA formulations achieved an Oxaliplatin (OXA) loading of 3.3 mg/mL. The complex hybrid nanosystem, CGNP+OXA+CSNP+RA, additionally showed a Retinoic Acid (RA) loading of 30.0 μg/mg.

**Figure 1:**
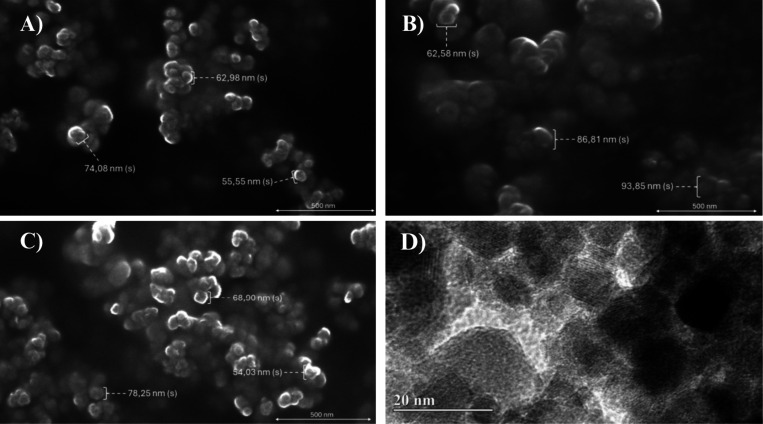
Morphological characterization of nanoparticles obtained by scanning electron microscopy (SEM) and transmission electron microscopy (TEM). Image (A) shows cashew gum nanoparticles (CGNP) at 500 nm magnification. Image (B) presents CGNP nanoparticles incorporated with Oxaliplatin (OXA), also at 500 nm magnification. Image (C) displays CGNP nanoparticles incorporated with OXA and retinoic acid (RA), functionalized with core–shell nanoparticles (CSNP), at the same magnification. Image (D) shows the morphology of CSNP obtained by TEM at 20 nm magnification.

### 2.2 In Vitro Oxaliplatin Release

Figure 2 shows a two-step OXA release pattern, which is common in polymer-based systems. The first phase indicates a rapid initial release (burst), followed by a sustained release. The initial phase corresponds to the release of the drug adsorbed on the nanoparticle surface. Subsequently, a relatively constant release occurs for up to 72 h, characterized by a process of drug diffusion through the nanoparticle structure. This sustained-release pattern proves promising for applications that require prolonged effects.

**Figure 2:**
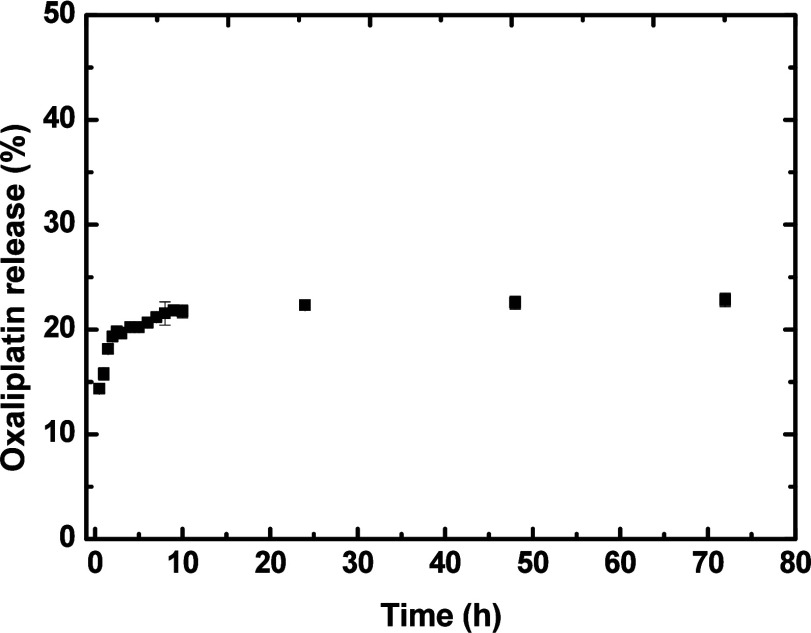
*In vitro* release profile of oxaliplatin from CGNP+OXA nanoparticle in PBS (pH = 7.4).

### 2.3 *In Vitro* Studies

#### 2.3.1 Cell Viability Tests

The cytotoxicity of oxaliplatin (OXA) was first evaluated at different concentrations after 24 and 48 h (Figure 3a). While OXA at 5–10 μg/mL did not significantly reduce cell viability, the 20 μg/mL treatment caused a marked reduction at 48 h (*p* < 0.05). The 25% DMSO control exhibited pronounced cytotoxicity at both time points (***p < 0.0001), confirming assay sensitivity. The effect of nanoparticle carriers was then investigated (Figure 3b). Core–shell nanoparticles (CS), cashew gum nanoparticles (CGNP), and their unloaded combinations did not significantly alter cell viability at 24 or 48 h, even under magnetic field (MF) exposure, indicating biocompatibility of the delivery systems. The main findings were observed with the OXA-loaded formulations (Figure 3c). Both CSNP+OXA+CGNP and CSNP+OXA+CGNP+RA significantly reduced tumor cell viability at 24 h (*p < 0.01) and remained effective at 48 h (*p* < 0.05) when compared to the DMEM control. Free OXA (10 μg/mL) also induced cytotoxicity at 24 h (*p* < 0.05), but its effect was not sustained at 48 h. In contrast, the CSNP+OXA+CGNP and CSNP+OXA+CGNP+RA formulations preserved their cytotoxic activity over time, highlighting their potential as more effective delivery systems for OXA.

**Figure 3:**
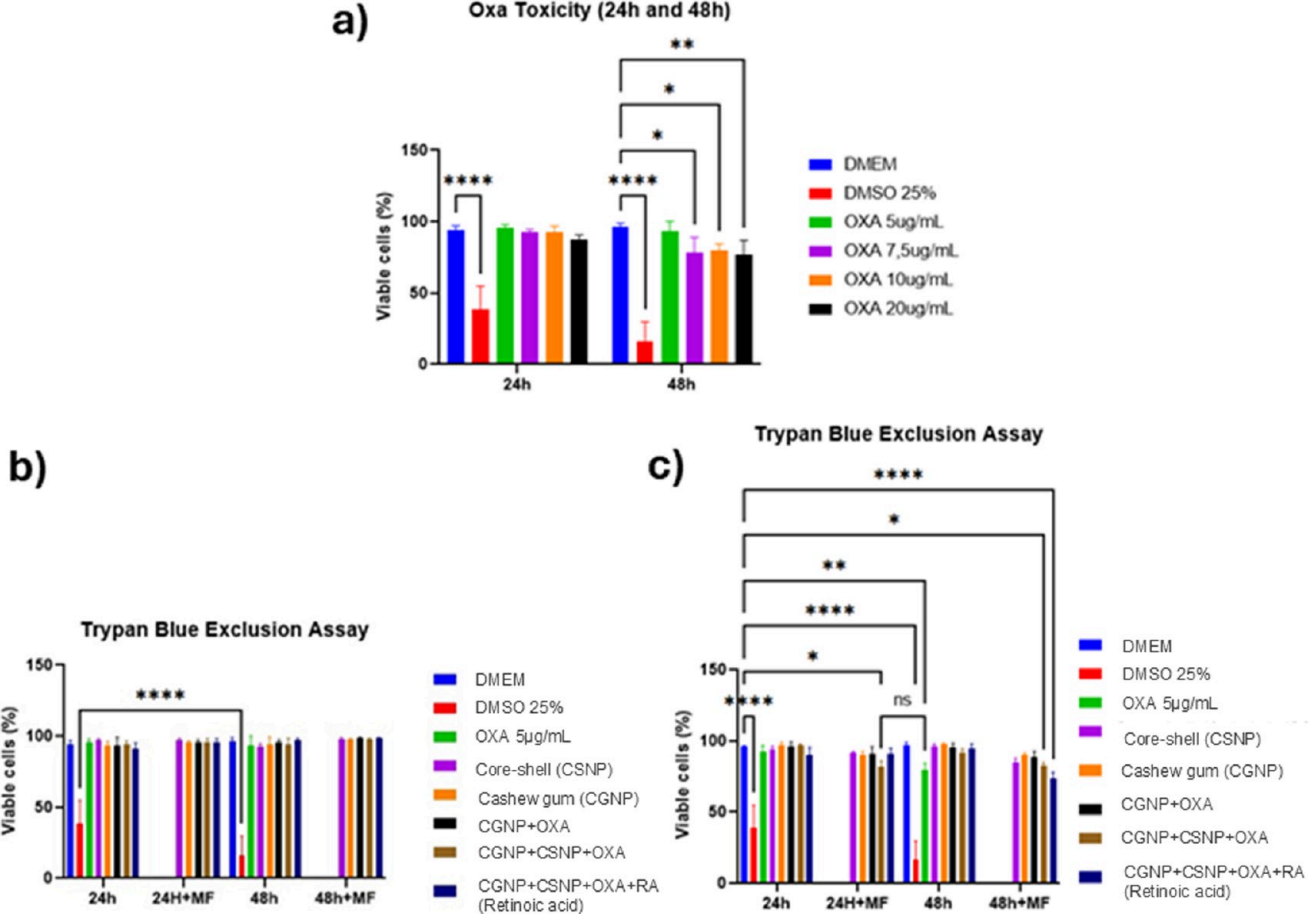
Cytotoxicity evaluation of oxaliplatin (OXA) and nanoparticle formulations in vitro. (a) OXA toxicity at different concentrations (5–20 μg/mL) after 24 and 48 h of incubation, assessed by Trypan Blue exclusion assay. (b) Viability of cells treated with nanoparticle formulations (Core–shell [CSNP], Cashew Gum [CGNP], CGNP+OXA, CGNP+CSNP+OXA, CGNP+CSNP+OXA+Retinoic Acid) with or without magnetic field (MF) exposure at 24 and 48 h. (c) Comparative cytotoxicity of OXA (10 μg/mL) and nanoparticle formulations under MF stimulation after 24 and 48 h. Data represent mean ± SD (*n* = 3). Statistical analysis was performed using two-way ANOVA followed by Bonferroni’s post hoc test (*p < 0.05; **p < 0.01; ***p < 0.001; ****p < 0.0001).

#### 2.3.2 Evaluation of the Cell Death Profile by Flow Cytometry

Flow cytometry analysis of cell death profiles in CT-26 cells, both in the absence and presence of a magnetic field, revealed distinct patterns of apoptosis induction. At 72 h, treatment with OXA significantly increased the proportion of early apoptotic cells compared to controls (*p < 0.05) (Figure 4A). Regarding late apoptosis, treatment with CGNP+OXA+CSNP+RA under magnetic field exposure led to a 143% increase compared to OXA alone (*p < 0.01), with OXA treatment alone also inducing a robust apoptotic response at both time points (****p < 0.0001). Notably, although not statistically significant, the group treated with CGNP+OXA+CSNP+RA under magnetic field exposure showed a 200% increase in late apoptosis at 48 h and a 70% increase at 72 h, compared to the same treatment without magnetic field exposure (Figure 4B). Additionally, after 72 h, the toxicity control group exhibited a significantly higher percentage of cell death compared to the OXA group (***p < 0.0001) (Figure 4C).

**Figure 4:**
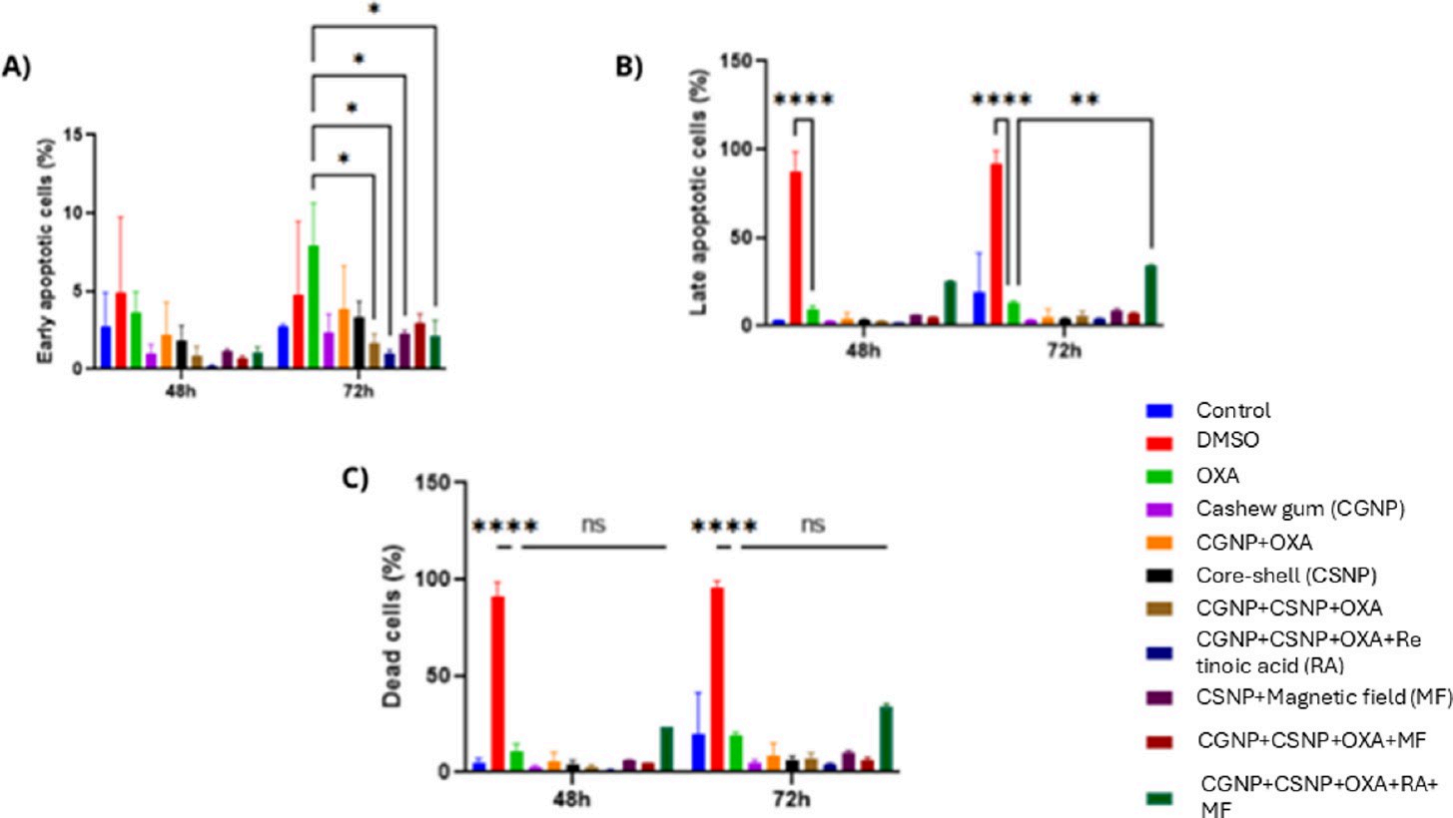
Cell death profile evaluated by flow cytometry. Statistical analyses present the percentage of early apoptotic cells at 48 and 72 h (A), late apoptotic cells at 48 and 72 h (B), and the overall cell death profile, including both apoptotic phases, at the same time points (C). Flow cytometry dot plots are provided as Supporting Information (Figures S1 and S2).

#### 2.3.3 Uptake Assay

Regarding the uptake of core–shell nanoparticles (CSNP), hybrid nanoparticles composed of cashew gum and oxaliplatin (CGNP+OXA+CSNP), and those further functionalized with retinoic acid (CGNP+OXA+CSNP+RA) by CT26 tumor cells, it was observed that CSNP were internalized by the tumor cells at both evaluated time points. Notably, a significantly higher internalization rate was observed after 48 h compared to 24 h (*p* < 0.05) (Figure 5). When comparing treated groups to the control (DMEM), a significantly greater nanoparticle uptake was observed in the CSNP group (**p < 0.001, ***p < 0.0001) and in the CGNP+OXA+CSNP+RA group (***p < 0.0001) at both time points (Figure 5C). Furthermore, the CGNP+OXA+CSNP+RA group exhibited a significantly higher internalization rate (***p < 0.0001) compared to the CSNP group (**p < 0.001). In contrast, the CGNP+OXA+CSNP group did not demonstrate a significant increase in internalization at either time point. Statistical analysis was performed using two-way ANOVA followed by Bonferroni’s post hoc test.

**Figure 5:**
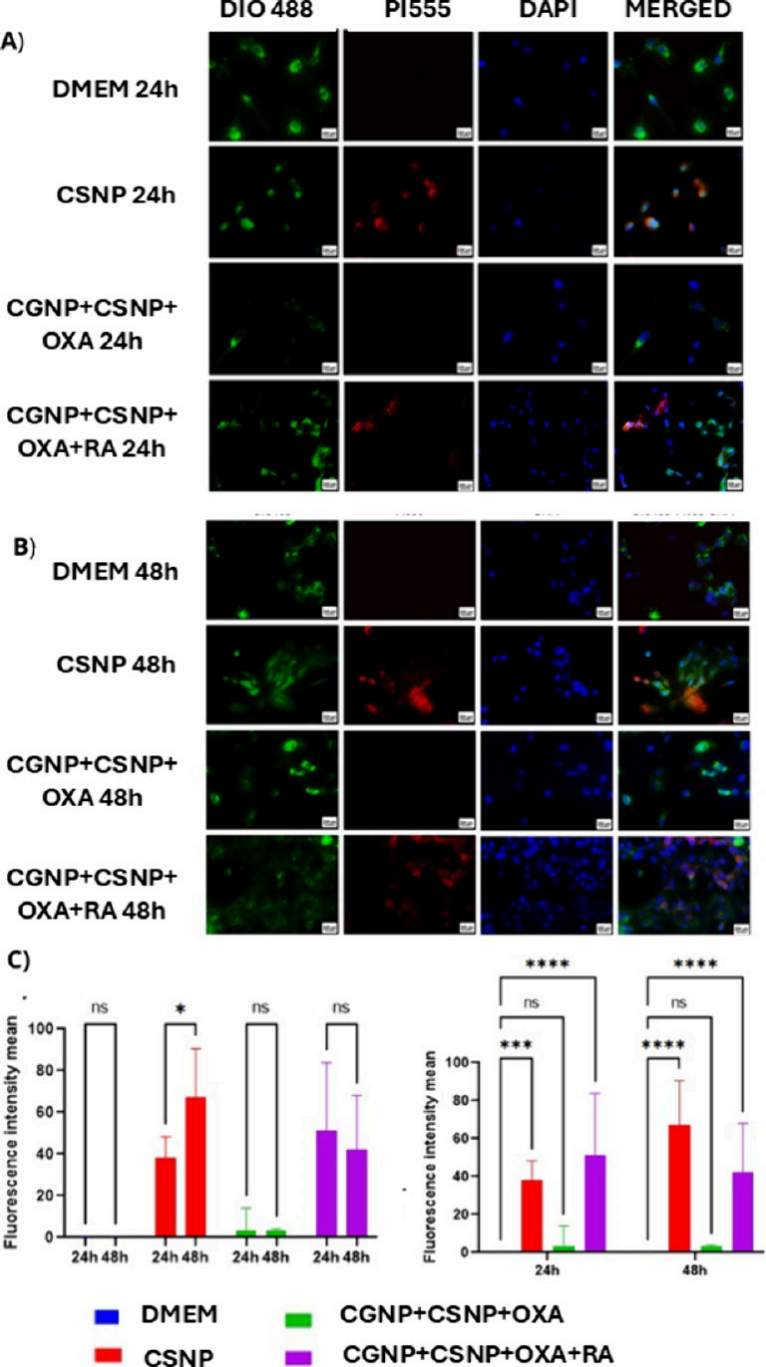
Cellular uptake analysis of nanosystems in CT26 tumor cells. A) Uptake of CSNP, CGNP+CSNP+OXA, and CGNP+ CSNP+OXA + RA groups after 24 h of treatment, using DiO, PI, and DAPI staining (microscopy, 200 μm scale). B) Uptake of the same compounds after 48 h of treatment under identical conditions. C) Quantification of mean fluorescence intensity, comparing uptake at 24 and 48 h for each nanosystem. A significant increase in cellular uptake was observed in the CSNP group at 48 h compared to 24 h (*p < 0.05). Statistical analysis was performed using two-way ANOVA followed by Bonferroni’s post hoc test.

### 2.4 In Vivo Antitumor Effects of Nanoparticles

#### 2.4.1 Assessment of Tumor Growth

In vivo tumor growth was assessed through macroscopic evaluation by measuring tumor area (mm^2^) throughout the treatment period (Figure 6C). No statistically significant differences in tumor growth were observed among the treated groups when compared to the control group.

**Figure 6:**
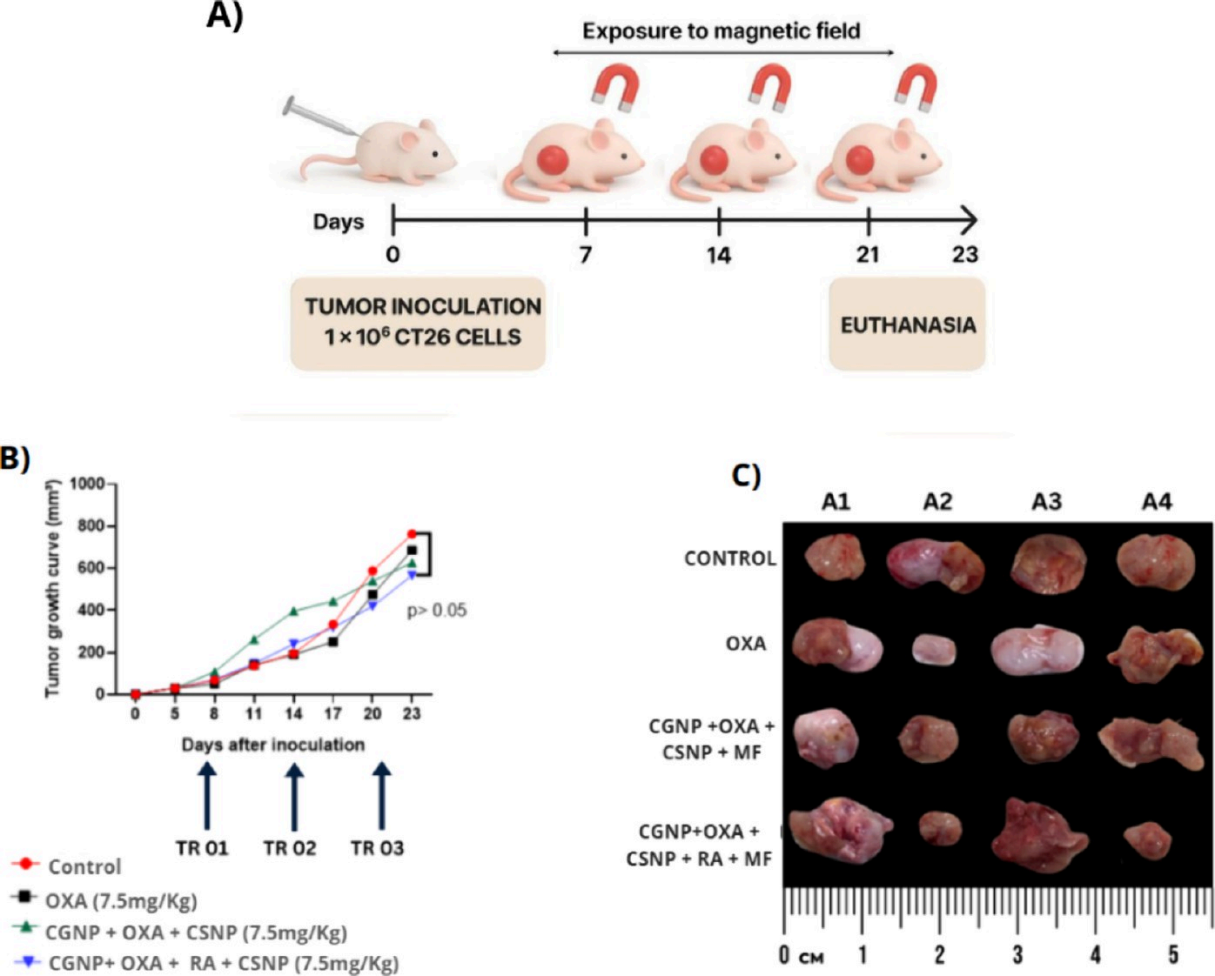
In vivo evaluation of combinatorial nanoparticle-based therapy in BALB/c mice. (A) Experimental timeline. BALB/c mice were subcutaneously inoculated with 1 × 10^6^ CT26 colorectal cancer cells on day 0. Treatments were administered on days 7, 14, and 21 (TR01–TR03), and animals were exposed to a magnetic field (MF) following each treatment. Euthanasia was performed on day 23.(B) Tumor growth curves for each experimental group: untreated control (red), oxaliplatin (OXA, 7.5 mg/kg; black), CGNP + OXA + CSNP + MF (green), and CGNP + OXA + CSNP + RA + MF (blue). Although a slight trend toward tumor growth inhibition was observed in the combinatorial therapy group, no statistically significant differences were found among groups (*p* > 0.05).(C) Ex vivo tumor images collected at the end point. Each row represents a treatment group (top to bottom: Control, OXA, CGNP + OXA + CSNP + MF, CGNP + OXA + CSNP + RA + MF), and columns A1–A4 correspond to individual animals (*n* = 4 per group). While there were visual variations in tumor sizes of some animals, the quantitative data presented in panel B confirms that tumor volume was consistent across the groups, with no statistically significant differences. Statistical analysis was performed using Kruskall-Wallis followed by Dunn’s post hoc test.

#### 2.4.2 Histopathological Analysis

Histopathological analysis revealed that treatment with the hybrid nano systems CGNP+OXA+CSNP and CGNP+OXA+CSNP+RA under magnetic field induction resulted in a significant reduction in the number of anaplastic cells (**p < 0.0001) and a marked increase in tumor necrotic areas (*p < 0.0001) (Figure 7). A decrease in inflammatory infiltrates was also observed in both treatment groups (**p < 0.001). In contrast, no significant changes in angiogenesis were detected in any of the nanoparticle-treated groups; however, treatment with Oxaliplatin alone led to a significant reduction in angiogenesis (**p < 0.001) (Figure 7E).

**Figure 7:**
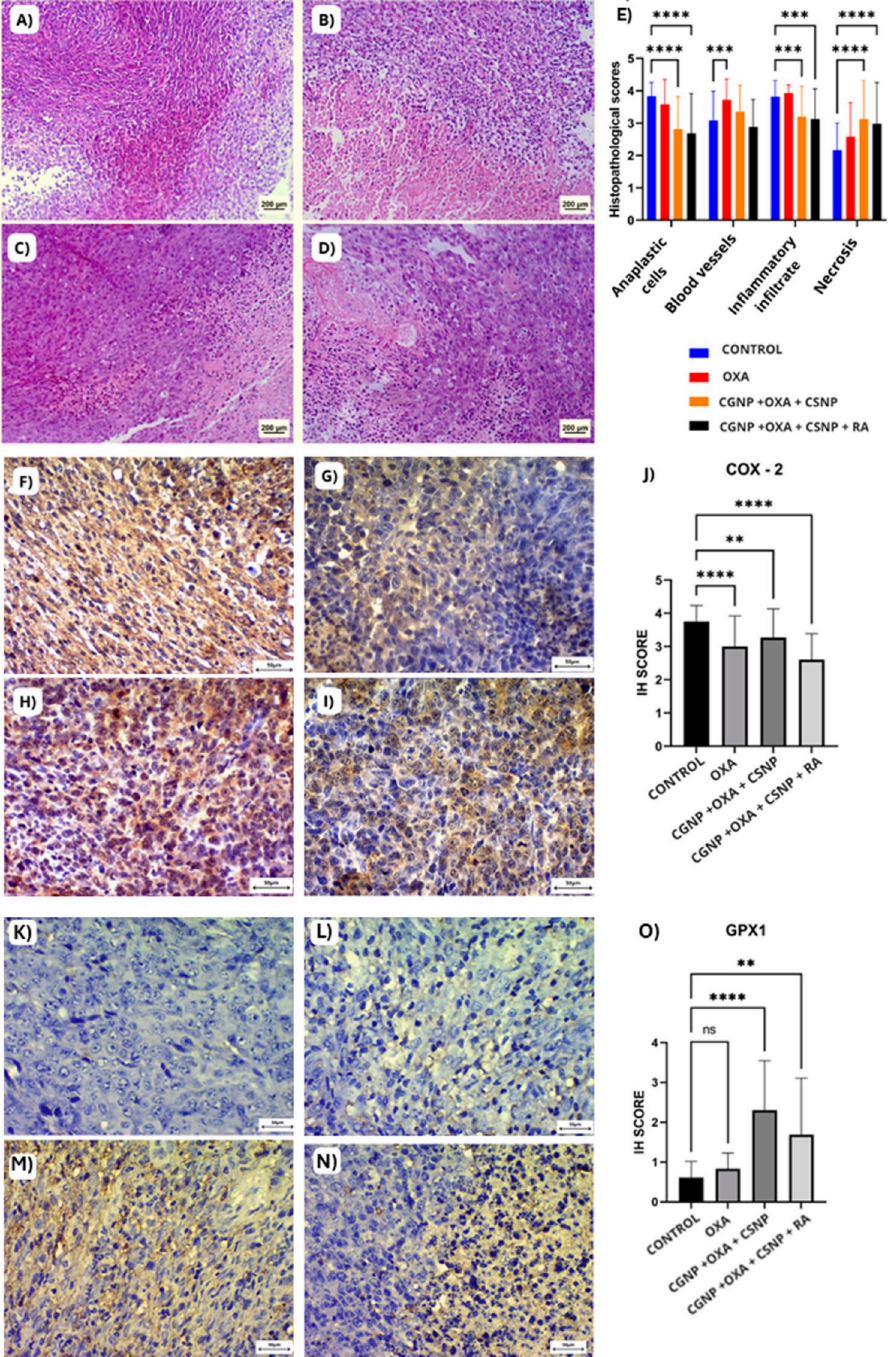
In vivo antitumor effects of nanoparticles. (A–D) Representative hematoxylin and eosin (H&E) staining showing histological features of tumors from each group: (A) Control, (B) OXA, (C) CGNP + OXA + CSNP, and (D) CGNP + OXA + CSNP + RA. Scale bar = 200 μm. (E) Histopathological scores for anaplastic cells, blood vessels, inflammatory infiltrate, and necrosis. (F–I) Immunohistochemistry (IHC) for COX-2 expression in tumor sections: (F) Control, (G) OXA, (H) CGNP + OXA + CSNP, and (I) CGNP + OXA + CSNP + RA. Scale bar = 50 μm. (J) Quantification of COX-2 immunoreactivity (H-score). (K–N) IHC for GPX1 expression: (K) Control, (L) OXA, (M) CGNP + OXA + CSNP, and (N) CGNP + OXA + CSNP + RA. Scale bar = 50 μm. (O) Quantification of GPX1 immunoreactivity (H-score). All histological and immunohistochemical images were acquired at 400× magnification. Statistical analysis was performed using Kruskall-Wallis followed by Dunn’s post hoc test. Data are expressed as mean ± SD. Statistical significance: **p < 0.01; ***p < 0.001; **p < 0.0001; ns = not significant.

#### 2.4.3 Detection of COX-2 and GPX-1 by Immunohistochemistry

Immunohistochemical analysis showed significant reductions in COX-2 expression in tumors treated with Oxaliplatin (**p < 0.0001), CGNP+OXA+CSNP (**p < 0.001), and CGNP+OXA+CSNP+RA (**p < 0.0001) compared to controls (Figure 7J), with the strongest effect observed in the Oxaliplatin and CGNP+OXA+CSNP+RA groups (Figure 7). Regarding GPX1, Oxaliplatin alone did not alter expression, whereas the combination treatment (CGNP+OXA+CSNP) significantly increased GPX1 levels, and RA maintained this effect without further enhancement (Figure 7O).

#### 2.4.4 Detection of SOD-1 and Hoechst 33342 by Immunofluorescence

The expression of the antioxidant enzyme superoxide dismutase (SOD-1) was evaluated to investigate the oxidative stress response in colorectal cancer cells. The control group exhibited high fluorescence intensity, while treatment with Oxaliplatin (OXA) resulted in a moderate reduction in fluorescence (*p* < 0.05) (Figure 8E). Although the CGNP+OXA+CSNP group displayed fluorescence levels comparable to the control, a statistically significant reduction in SOD activity was observed (*p* < 0.05). Notably, the CGNP+OXA+CSNP+RA group exhibited a pronounced decrease in fluorescence intensity, indicating a substantial reduction in SOD activity or expression (*p < 0.01) (Figure 8E). In addition, Hoechst 33342 staining was employed to characterize the type of cell death induced by the nano systems. In the OXA group, fluorescence patterns consistent with necrotic cell death were observed, including diffuse staining and loss of nuclear integrity, indicative of membrane rupture and irregular DNA distribution. In the CGNP+OXA+CSNP group, fluorescence intensity ranged from moderate to high and was comparable to that of the control group. Conversely, cells treated with the fully functionalized nano system (CGNP+OXA+CSNP+RA) exhibited significantly higher fluorescence intensity, with clear nuclear boundaries, suggesting increased apoptotic activity. This difference was statistically significant compared to the control group (**p < 0.0001) (Figure 8J).

**Figure 8:**
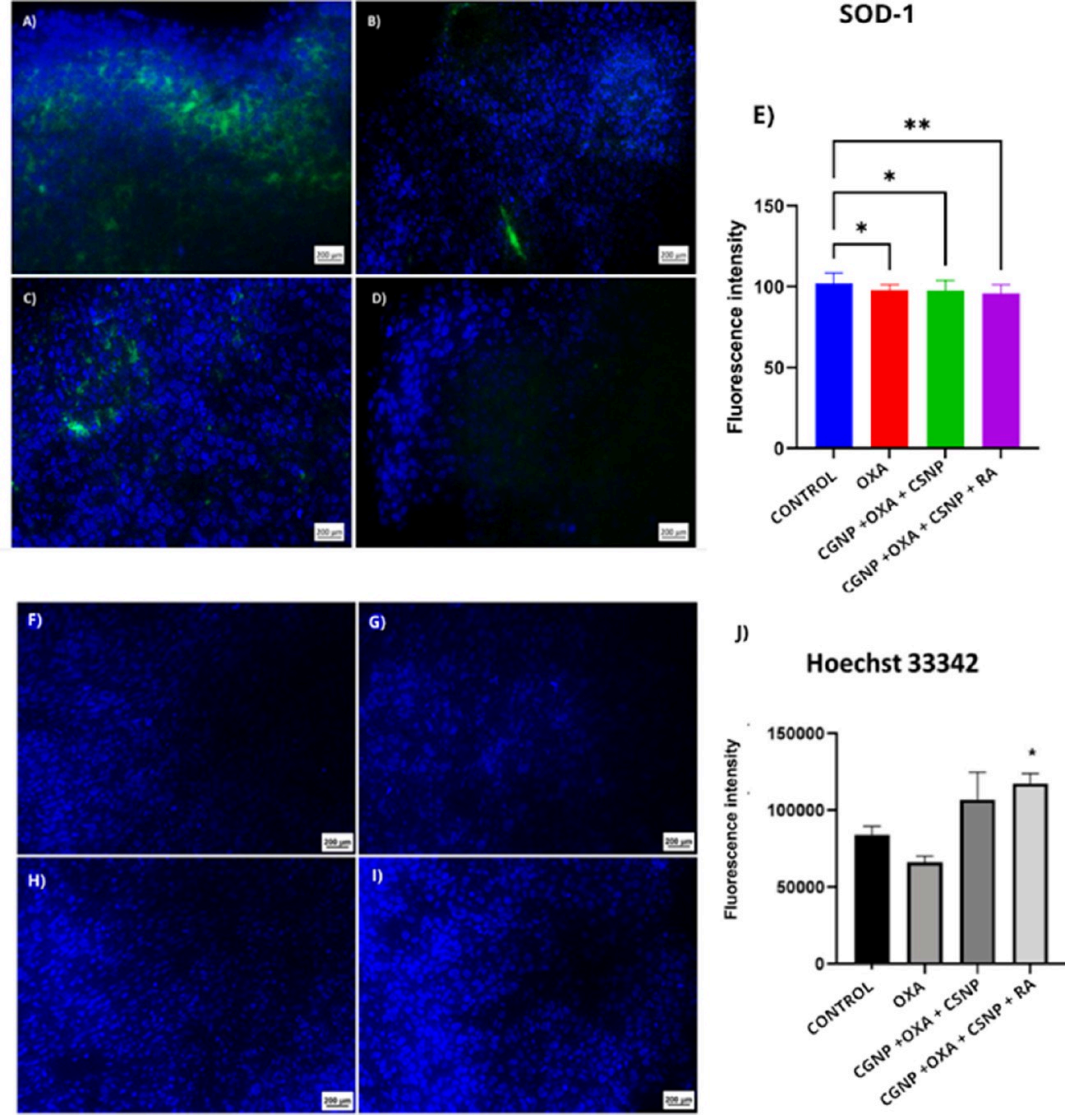
Immunofluorescence analysis. (A) Saline group displaying intermediate to high fluorescence intensity for SOD-1. (B) OXA group showing intermediate SOD-1 fluorescence. (C) CGNP+OXA+CSNP group with high SOD-1 fluorescence intensity. (D) CGNP+OXA+CSNP+RA group exhibiting low SOD-1 fluorescence intensity. (E) Quantitative analysis comparing SOD-1 immunolabeling among treated groups and the control (saline). (F) Saline group with intermediate fluorescence intensity for Hoechst 33342. (G) OXA group showing low Hoechst 33342 fluorescence. (H) CGNP+OXA+CSNP group presenting fluorescence intensity ranging from intermediate to high, similar to the saline group. (I) CGNP+OXA+CSNP+RA group exhibiting high fluorescence intensity for Hoechst 33342. (J) Quantitative analysis of Hoechst 33342 fluorescence comparing treated groups with the control group (saline). All images captured at 630× magnification.

## 3 Discussion

In this study, the nanosystem composed of cashew gum-based nanoparticles (CGNP) loaded with oxaliplatin (OXA) and retinoic acid (RA), combined with magnetically responsive core–shell nanoparticles (CSNP), proved capable of enhancing chemotherapeutic delivery, improving cellular uptake, and amplifying cytotoxic and apoptotic responses in colorectal cancer models. DLS and Zeta potential analyses on the CSNP samples (Table 1) confirmed the isolated Core–Shell Nanoparticles to be highly aggregated and heterogeneous, yielding a final microparticle size (1699.6 ± 520.6 nm) and high Polydispersity Index (PDI = 0.40 ± 0.3). This result likely reflects transient aggregation detected by DLS, which is highly sensitive to small populations of larger clusters. In contrast, TEM confirmed that CGNP+CSNPs+OXA+RA formulation was nanosized (∼70 nm, Figure 1C). The CSNP suspensions showed limited colloidal stability and partial sedimentation, consistent with the magnetic nature of iron oxide nanoparticles and their tendency to form reversible aggregates via dipole–dipole interactions.^10,11^ Once incorporated into the cashew-gum matrix (CGNP + OXA + CSNP + RA), the particles formed stable and homogeneous dispersions without visible sedimentation or color change. This stabilization arises from electrostatic and hydrogen-bonding interactions between polysaccharide functional groups and the metallic surface, as well as steric barriers that prevent magnetic clustering.^10,11^ The negative zeta potential (−36 ± 0.2 mV) of the hybrid nanosystem supports this effect, indicating strong electrostatic repulsion. Therefore, the large DLS size represents reversible agglomeration in aqueous medium rather than true micrometric dimensions, while incorporation into the cashew-gum network yielded a stable nanosystem (∼200 nm, DLS-measured size) suitable for biomedical use.

Gold plays a crucial role in CSNPs for the delivery of anticancer drugs by enhancing drug stability, enabling controlled release, and facilitating targeted delivery. These nanoparticles leverage the unique properties of gold to improve the efficacy and safety of cancer treatments. Gold nanoparticles (AuNPs) are used to encapsulate anticancer drugs, like doxorubicin, within core–shell structures, which stabilizes the drug, prevents rapid diffusion, and allows for sustained release.^12^ The photothermal properties of gold also enable controlled drug release triggered by near-infrared (NIR) light.^12^ Gold-coated iron nanoparticles are utilized for magnetic drug targeting, where the magnetic core concentrates the nanoparticles at the tumor site, enhancing local drug concentration and reducing systemic exposure.^13^ Furthermore, the functionalization of AuNPs with targeting ligands, such as folic acid, improves the precision of delivery to specific cancer cells.^14^ As a biocompatible material, gold’s surface can be modified to improve drug loading and conjugation with other therapeutic agents.^15^ The combination of gold with other materials, like PEGylated iron oxide, can also enhance the therapeutic effects of drugs by increasing their cytotoxicity against cancer cells.^16^ While these systems offer significant advantages, challenges such as potential toxicity, cost, and scalability remain, requiring further research for clinical optimization.

Our development of a multifunctional nanosystem to enhance the delivery and efficacy of oxaliplatin (OXA) for colorectal cancer, particularly by transitioning cell death to controlled apoptosis, reflects a critical trend in precision oncology. The core–shell design and encapsulation strategy address the challenge of systemic toxicity associated with heavy metal-based chemotherapeutics, such as the Pt-derived OXA. This rationale is strongly corroborated by Lai et al. (2025), who developed a Zn­(II)-Schiff base complex and explicitly highlighted the systemic risks of transition metal complexes (e.g., Ir­(III), Ru­(II), and conventional Pt drugs), advocating for inherently biocompatible cores like zinc to build safer ″track-and-treat″ theranostics.^17^ Therefore, the successful in vivo modulation of the tumor microenvironment and induction of apoptosis with our hybrid platform validate the imperative of combining biocompatible carriers with enhanced delivery mechanisms to shift therapeutic outcomes toward regulated cell death, a necessity for advancing cancer therapy beyond conventional toxic regimens.

Building on the foundation of a gold-based drug delivery system, the therapeutic efficacy of this approach was confirmed through cell viability assays. As demonstrated, a dose-dependent decrease in CT-26 cell viability was observed after exposure to free OXA, and 10 μg/mL was identified as the optimal concentration for further experiments (Figure 3). Notably, the combined formulation CGNP+OXA+CSNP+RA under magnetic field exposure led to the most substantial viability reduction, suggesting a possible synergistic interaction­(Figure 3c). Although the exact mechanisms are not entirely understood, previous studies have shown that all-trans retinoic acid (RA) modulates Wnt/β-catenin signaling by downregulating β-catenin, c-MYC, and cyclin D1, while upregulating E-cadherin, promoting apoptosis and differentiation.^3,18^ It is also known that RA may suppress oncogenic microRNAs such as miR-200a-3p and miR-141–3p,^3,19^ which could further explain the enhanced cytotoxicity observed in the combination therapy.

Flow cytometry analysis (Figure 4) showed a 200% increase in late-stage apoptosis in the CGNP+OXA+CSNP+RA group under magnetic field stimulation, compared to the same treatment without magnetic exposure. This effect may be attributed to the increased intracellular accumulation and more effective activation of apoptotic pathways. RA induces apoptosis primarily through RAR/RXR signaling, leading to the downstream expression of pro-apoptotic proteins like caspase-3, BAX, and FADD, while reducing antiapoptotic markers such as BCL-2.^3^ When coencapsulated with OXA in PLGA nanoparticles, RA has been shown to enhance cytotoxic effects in colorectal cancer cells by amplifying apoptosis both in vitro and in vivo.^20^ These effects are further intensified under magnetic stimulation, as magnetic fields promote localized hyperthermia and increase intracellular ROS, contributing to lysosomal disruption and mitochondrial damage, thereby reinforcing apoptotic activity.^21^ Additionally, previous studies show that magnetic guidance allows for more precise drug accumulation at the tumor site and enables controlled RA and OXA release, enhancing efficacy and reducing systemic toxicity.^22–24^

These apoptotic effects appear closely linked to cellular uptake (Figure 5). The enhanced apoptosis observed with CGNP+OXA+CSNP+RA correlates with its higher internalization at 24 h, although uptake declined relative to CGNP+OXA+CSNP at 48 h. This apparent reduction likely reflects the biological action of retinoic acid (RA), which promotes early uptake but also accelerates apoptosis, as evidenced by the marked increase in late apoptotic cells at 48–72 h. This mechanism has long been documented in the literature, as cells undergoing advanced apoptosis progressively lose endocytic capacity, explaining the diminished signal at later time points.^25^ In contrast, CGNP+OXA+CSNP-treated cells remained more viable, sustaining uptake over time. Thus, the 48 h difference reflects earlier cytotoxic/apoptotic onset in the RA-functionalized system rather than reduced uptake efficiency. Beyond its cytotoxic role, RA may also influence receptor-mediated endocytosis through nuclear receptor signaling (RAR/RXR), modulating genes involved in adhesion, migration, and immune regulation, thereby contributing to the overall therapeutic effect.^26,27^

Despite promising in vitro findings, tumor volume did not significantly differ across treatment groups in vivo (Figure 6B,C). This divergence may reflect physiological limitations such as suboptimal release, poor tumor penetration, or the short duration of treatment. Tumor volume measurements, although standard, may not fully capture treatment efficacy, especially when necrosis or other molecular effects are not accompanied by shrinkage. As discussed by Goldmacher and Conklin (2011), tumor volumetrics can mask therapeutic necrosis or active resistance, which calls for complementary imaging methods—such as 18F-FDG PET, 18F-FLT PET, diffusion-weighted MRI, and perfusion imaging—to better assess therapeutic outcomes.^28^

Histopathological analysis (Figure 7A–E) showed significant antitumor effects, including reduced anaplastic cells and increased tumor necrosis, even without marked tumor volume reduction. Both the CGNP+OXA+CSNP and CGNP+OXA+CSNP+RA treatments under magnetic induction were effective. The treatments also reduced inflammatory infiltrates, likely due to immunomodulatory activity and COX-2 suppression. Immunohistochemical analysis confirmed this (Figure 7f–j), with significant reductions in COX-2 expression in the OXA, CGNP+OXA+CSNP, and CGNP+OXA+CSNP+RA groups. The RA-functionalized nanosystem showed the most pronounced effect, suggesting RA enhances oxaliplatin’s anti-inflammatory and antiproliferative properties by modulating COX-2.^20^

Immunohistochemical analysis also revealed increased levels of GPx-1­(Figure 7K–O), particularly in the CGNP+OXA+CSNP group, indicating a possible adaptive antioxidant response to therapy-induced oxidative stress.^29^ This response, however, was insufficient to counteract the oxidative imbalance, a conclusion supported by other findings such as increased apoptosis. Thus, the elevation of GPx-1 serves as an indicator that the treatment effectively induces oxidative stress, ultimately overwhelming tumor defenses and enhancing apoptosis.^29^ Furthermore, immunofluorescence staining for SOD-1 showed a marked decrease in the CGNP+OXA+CSNP+RA-treated group, suggesting that RA contributes to redox homeostasis disruption, potentially intensifying cytotoxicity. As previously reported, retinoids can suppress antioxidant defenses and increase oxidative damage by downregulating the Keap1/Nrf2/ARE axis.^3^ Together, these results suggest that the nanosystem not only downregulates pro-inflammatory mediators but also modulates redox homeostasis, enhancing tumor susceptibility to oxaliplatin.

Moreover, these findings align with the mechanism of magnetic-induced hyperthermia, where alternating magnetic fields (AMFs) raise local temperatures to 43–46 °C and generate reactive oxygen species (ROS), inducing apoptosis or necrosis via organelle disruption.^21^ However, it is important to note that, in our experimental setup, the thermal monitoring system did not exhibit sufficient sensitivity to detect measurable temperature variations at the low concentrations of CSNPs employed in this study (10 μg/mL for in vitro assays and 7.5 mg/kg for in vivo experiments). Despite this limitation, the observed biological effects under magnetic field stimulation suggest that mechanisms such as mechanical vibration of nanoparticles and ROS generation may contribute to the therapeutic outcomes independently of detectable bulk heating. The synergy between magnetic stimulation and chemotherapeutic agents thereby amplifies cytotoxicity and improves therapeutic efficacy.^22^

Interestingly, a significant reduction in angiogenesis was only seen in the free OXA group. This was unexpected, as nanoparticle encapsulation is generally expected to enhance drug delivery. A possible explanation is that the controlled release of OXA from the CGNP systems resulted in insufficient drug concentration in endothelial cells. Alternatively, sustained or continuous exposure to OXA may be necessary to inhibit angiogenesis effectively. Supporting this, Liang et al. (2018) demonstrated that encapsulation of an oxaliplatin prodrug (Oxa­(IV)) within mesoporous silica nanoparticles functionalized with D-α-tocopheryl polyethylene glycol 1000 succinate (TPGS)—a water-soluble derivative of vitamin E used as a surfactant and drug efflux inhibitor—enabled sustained and self-regulated drug release.^30^ The TPGS coating acted as a steric barrier, maintaining the prodrug in an inactive form until reduction inside tumor cells, thereby allowing spatial and temporal control over release. Although angiogenesis was not directly evaluated in that study, the sustained release profile observed may help explain the lack of antiangiogenic effects in our nanoformulations due to limited endothelial exposure.^30^

Hoechst 33342 nuclear staining (Figure 8) made it possible to distinguish between the various experimental groups’ modes of cell death. In contrast to cells exposed to the CGNP+OXA+CSNP+RA formulation, which showed strong fluorescence and well-defined nuclei with chromatin condensation indicative of apoptosis, cells treated with free oxaliplatin (OXA) showed diffuse nuclear fragmentation, which is consistent with necrosis. Crowley et al. (2016) states that Hoechst 33342 staining works well for differentiating between apoptotic cells, which show nuclear condensation and fragmentation, and healthy or necrotic cells, whose nuclei typically have more uniform staining and an intact morphology.^31^ These findings are supported by previous studies demonstrating that apoptosis is associated with DNA fragmentation and chromatin compaction,^32–34^ while such alterations are typically absent in necrosis. Furthermore, the morphological analysis correlated strongly with flow cytometry data, which revealed a substantial increase in late apoptotic cells in the CGNP+OXA+CSNP+RA group under magnetic field exposure. Collectively, these results indicate that the RA-functionalized nanosystem preferentially induces regulated apoptotic pathways rather than necrotic cell death. This distinction has important clinical implications, as apoptosis is a controlled process that prevents the release of pro-inflammatory intracellular contents, thereby preserving surrounding tissues and contributing to a safer therapeutic profile.^35,36^

Altogether, the findings support a complex antitumor mechanism for the CGNP+OXA+CSNP+RA system, involving enhanced cellular uptake, apoptotic induction, COX-2 suppression, and oxidative stress exacerbation. Future research should focus on biodistribution analysis, drug release profiling, and in-depth evaluation of tumor penetration to further optimize this nanoplatform. Although no signs of acute systemic toxicity (mortality or behavioral alterations) were observed, detailed analyses of body weight, serum biochemistry, and histology of major organs were not performed. Future work will include systematic evaluation of ALT, AST, creatinine, and BUN levels, along with histopathological assessment of liver, kidney, spleen, and heart tissues to fully establish the biocompatibility profile of the nanosystem.

Beyond its individual therapeutic potential, the CGNP+OXA+CSNP+RA nanosystem shows promise as a versatile platform for targeted cancer therapy and as a combinatory approach with immunotherapies. The magnetic responsiveness and RA-mediated uptake enhance site-specific delivery — an essential feature for precision oncology. Moreover, the observed reduction in inflammatory infiltrates and COX-2 expression suggests that this system may modulate the tumor immune microenvironment, potentially improving the efficacy of immune checkpoint inhibitors or dendritic cell-based vaccines. Future investigations integrating this nanosystem with immune-modulating agents, such as anti-PD-1/PD-L1 antibodies or cytokine adjuvants, could unveil synergistic interactions and pave the way for multimodal therapeutic strategies against colorectal and other solid tumors.

## 4 Material and Methods

### 4.1 Materials

The following reagents were used in cell-based experiments: Dulbecco’s Modified Eagle Medium (DMEM; Life Technologies, Grand Island, NY, USA), heat-inactivated fetal bovine serum (FBS; Cultilab, Campinas, Brazil), trypsin/EDTA (Gibco, Life Technologies), oxaliplatin (Eurofarma, São Paulo, Brazil), CGNPs (provided by UFC, Fortaleza, Brazil), CSNPs (Institute of Chemistry, UFRN, Natal, Brazil), sodium hydroxide, glycerol, and polyvinylpyrrolidone (Sigma-Aldrich), and the Dead Cell Apoptosis Kit (Invitrogen, Waltham, MA, USA), Hoechst 33342 staining (H1399, Invitrogen, Waltham/MA, USA).

### 4.2 Nanoparticles Synthesis and Characterization

#### 4.2.1 CGNP Synthesis and Characterization

The synthesis of cashew gum nanoparticles (CGNP) followed a previously established protocol.^37^ Initially, 1 g of cashew gum (CG) was dissolved in dimethyl sulfoxide (DMSO) at 70–75 °C under continuous magnetic stirring. Following dissolution, l-lactide and triethylamine (TEA) were introduced into the reaction mixture, which was maintained under a nitrogen atmosphere for 2 h before discontinuing the gas flow. The reaction was then allowed to proceed for an additional 10 h. After completion, the resulting copolymer was filtered, dialyzed against water, and lyophilized. The grafting reaction was performed at two different molar ratios of CG to PLA (1:1 and 1:10), with fixed concentrations of l-lactide (10% w/v) and TEA (2% w/v). To eliminate residual monomers and potential homopolymers, hexane was added to the lyophilized grafted copolymers dispersed in distilled water, and the mixture was stirred magnetically for 48 h. The aqueous phase was subsequently separated and lyophilized. The nanoparticles were characterized for particle size, polydispersity index (PDI), and zeta potential using dynamic light scattering (DLS) with a 633 nm laser at a 173° scattering angle. All measurements were conducted in triplicate at room temperature. For morphological analysis, scanning electron microscopy (SEM) was employed. Samples were prepared by depositing 10 μL of each nanoparticle suspension onto carbon tape, followed by drying at ambient temperature.

#### 4.2.2 CSNP Synthesis and Characterization

The synthesis of magnetic nanoparticles - core–shell nanoparticles (CSNPs) - began with the preparation of spherical gold nanoparticles (AuNPs, 7.1 nm diameter),^38^ where all glassware was first cleaned by immersion in a KMnO_4_ + NaOH solution overnight, rinsed with deionized water, and treated with a 1:1 (v/v) H_2_O_2_ + H_2_SO_4_ solution for 10 min before final rinsing and drying. For AuNP synthesis, 0.20 g of polyvinylpyrrolidone (PVP) and 6.80 mg of gold chloride (AuCl_3_) were dissolved in 10 mL of deionized water, while a separate solution containing 0.18 g of glycerol and 0.080 g of NaOH in 10 mL of deionized water was prepared and then combined with the AuCl_3_–PVP mixture, resulting in final concentrations of 1.0 mmol·L^–1^ Au^3^^+^, 0.10 M NaOH, 0.10 M glycerol, and 10 g·L^–1^ PVP, with successful AuNP formation indicated by a dark red color. The AuNPs were characterized by UV–visible spectroscopy (Evolution 60S spectrophotometer, Thermo Scientific, USA) and fluorescence spectroscopy (RF-5301 PC fluorophotometer, Shimadzu, Japan). To synthesize the CSNPs, AuNPs were deposited onto a magnetite (Fe_3_O_4_) core prepared by mixing 5 mL each of PVP (5.0 g·L^–1^), FeCl_2_ (0.40 mol·L^–1^), and FeCl_3_ (0.80 mol·L^–1^) solutions under magnetic stirring, followed by addition of 5 mL of KOH (3.2 mol·L^–1^) to form a 20 mL black suspension confirming Fe_3_O_4_ formation. A 2 mL aliquot of this suspension was diluted with 8.0 mL of deionized water, and 1000 μL of the AuNP solution (10 mmol·L^–1^) was added under stirring to form CSNPs, which were subsequently characterized by UV–visible spectroscopy (Evolution 60S spectrophotometer).

#### 4.2.3 Hybrid CGNP and CSNP Emulsion Synthesis and Characterization

The emulsions containing oxaliplatin-loaded core–shell nanoparticles (CSNPs), cashew gum nanoparticles (CGNP), and retinoic acid (RA) hybrid systems were prepared following the methodology described by Calvo et al. (1997).^39^ The aqueous phase was formulated using a 10 mg/mL solution of hydrophobically modified cashew gum (CGProp) dissolved in DMSO and deionized water, with the final volume adjusted to 20 mL. Immediately after preparing the aqueous phase, the organic phase was introduced, consisting of 0.5 mL ethanol, 125 μL Miglyol, and 9.5 mL acetone. The mixture was then processed using a rotary evaporator (IKA RV3 eco) at 45 °C to concentrate the solution by removing ethanol, acetone, and excess water until approximately one-third of the original volume remained. The resulting emulsions were isolated through centrifugation at 15,000 rpm for 1 h at 25 °C using a Hettich EBA 21 centrifuge. All isolated emulsion samples were subsequently stored under refrigerated conditions at 4 ± 2 °C for further analysis. For nanoparticle characterization, key parameters including particle size, polydispersity index (PDI), and zeta potential were determined by dynamic light scattering (DLS) measurements. These analyses were performed using a 633 nm laser at a fixed scattering angle of 173°. Prior to measurement, 50 μL aliquots of each emulsion sample were suspended in 1 mL of deionized water and analyzed immediately after the centrifugation-based isolation process. Four distinct formulations were developed and evaluated in this study. The base formulation (F1) consisted of 2 mL of hydrophobized cashew gum (10 mg/mL) combined with 18 mL deionized water. Formulation F2 incorporated oxaliplatin (OXA) by adding 0.3 mL of OXA solution (3.3 mg/mL) to the base components, with the water volume adjusted to 17.7 mL. The third formulation (F3) included both OXA and CSNPs, with 1 mL of core–shell nanoparticles (containing 0.74 mmol Au and 2 mL Fe) added to the mixture, requiring a reduction of water volume to 16.5 mL. The most complex formulation (F4) combined all components: the base cashew gum solution, 20 mg retinoic acid (RA), 0.5 mL OXA solution, 1 mL CSNPs, and 16.5 mL deionized water.

#### 4.2.4 In Vitro Oxaliplatin Release Assay

Oxaliplatin (OXA) release study from CGNP+OXA nanoparticle was performed in phosphate buffer (PBS; pH = 7.4; 0.1 mol L^–1^) containing sodium lauryl sulfate (LSS; 0.1% m/v) by the dialysis method. Briefly, the nanoparticle (1 mL) was dispersed in 1 mL of PBS and transferred to a cellulose membrane (cut off 14,000 g mol^–1^). The membrane was then immersed in PBS and incubated at 37 °C for 72 h in the dark with constant shaking (75 rpm). At predetermined time intervals, 1 mL of release medium was collected and replaced with an equal volume of fresh buffer. The released OXA was analyzed by HPLC at 240 nm.

### 4.3 Development and Application of the Magnetic Field Induction System

To activate the CSNPs, a magnetic field induction system was constructed following protocols previously stablished.^7^ The setup, developed at the Federal University of Rio Grande do Norte (UFRN), produces alternating magnetic fields up to 250 Oe and 200 kHz, detecting temperature changes as low as 0.1 °C from −30 to 500 °C. The system includes a signal generator (Agilent 33250A), a power amplifier (SKP 1220X), and LC resonant circuits with copper solenoids (Supporting Information file, Figure S3). A Minipa MO-1222 oscilloscope tuned the resonance frequency. The thermal monitoring system included a sample holder inside the solenoid, an Teledyne FLIR Extech HD300 infrared thermometer connected to a computer with the Teledyne FLIR Extech HF300 2.3 software installed, and a closed-loop cooling system using a hydraulic pump and chiller. A custom-designed solenoid tailored to fit 60 × 15 mm Petri dishes was employed for both in vitro and in vivo applications (Figure S3f,g).

For magnetic stimulation, Petri dishes containing CT-26 cells or anesthetized mice were positioned at the center of the solenoid, ensuring optimal exposure to the magnetic field. The system was set up to generate a 50 kHz field at 20 V, which is safe for biological systems while sufficient to induce magnetic nanoparticle vibration and trigger oxaliplatin release.^14–17^ Each exposure lasted 3 min, followed by a 5 min cooling interval between sessions to prevent device overheating and ensure the thermal safety of the biological samples.

### 4.4 In Vitro Cellular Studies

#### 4.4.1 Cell Culture

CT-26 murine colon adenocarcinoma cells (ATCC CRL-2638) were cultured in DMEM supplemented with 10% FBS, maintained at 37 °C in a humidified incubator with 5% CO_2_. The culture medium was replaced three times per week, and phosphate-buffered saline (PBS) was used to wash the cells before each replacement.

#### 4.4.2 Cell Viability

Cell viability was assessed using the Trypan Blue exclusion method.^40^ Cells were seeded in duplicate into 12-well plates (0.1 × 10^6^ cells/well) and treated for 24 or 48 h with the following formulations: DMEM (control), 25% DMSO (toxicity control), OXA (5, 7.5, 10, 20 μg/mL), CSNP (5 and 10 mM/mL), CGNP (5 and 10 μg/mL), CGNP + OXA (5 and 10 μg/mL), CGNP + OXA + CSNP (5 and 10 μg/mL), and CGNP + OXA + CSNP + RA (5 and 10 μg/mL). To evaluate magnetic field (MF) effects, cells were plated in 60 × 15 mm Petri dishes (3 × 10^5^ cells/dish) and treated with the same formulations for 24 or 48 h. After treatment, dishes were positioned inside the solenoid and exposed to a 50 kHz, 20 V magnetic field for 3 min. Cells were harvested with trypsin/EDTA, neutralized with DMEM, centrifuged at 3500 rpm for 5 min, stained 1:1 with 0.4% Trypan Blue, and counted using a Neubauer chamber. Viability (%) was calculated using the formula: Viability = [1 - (number of stained cells/total cells)] × 100. Statistical significance was assessed via Kruskal–Wallis followed by Dunn’s post hoc test. Graphs and analyses were generated using GraphPad Prism v9.0.0.

#### 4.4.3 Flow Cytometry

Flow cytometry was used to determine apoptosis levels. Cells not exposed to the magnetic field were plated in 6-well plates (2 × 10^5^ cells/well), and MF-treated cells were plated in 60 × 15 mm Petri dishes (3 × 10^5^ cells/dish). Treatments were applied for 48 or 72 h according to Table 2.

**Table 2:** Description of Experimental Groups Used in Flow Cytometry Assays, with Corresponding Treatments, Formulations, and Concentrations^a^

Group	Abbreviation	Description	Concentration
1	DMEM	No treatment	-
2	DMSO	Toxicity control	25%
3	OXA	Oxaliplatin	10 mg/mL
4	CGNP	Cashew gum nanoparticles	10 μg/mL
5	CGNP + OXA	Cashew gum nanoparticles functionalized with oxaliplatin	10 μg/mL
6	CSNP	Core–shell nanoparticles	10 μM/mL
7	CGNP + OXA + CSNP	Cashew gum nanoparticles functionalized with oxaliplatin, hybridized with core–shell nanoparticles	10 μg/mL
8	CGNP + OXA + CSNP + RA	Cashew gum nanoparticles functionalized with oxaliplatin and retinoic acid, hybridized with core–shell nanoparticles	10 μg/mL
9	CSNP + MF	Core–shell nanoparticles and magnetic field exposure	10 μM/mL
10	CGNP + OXA + CSNP + MF	Cashew gum nanoparticles functionalized with oxaliplatin, hybridized with core–shell nanoparticles and magnetic field exposure	10 μg/mL
11	CGNP + OXA + CSNP + RA + MF	Cashew gum nanoparticles functionalized with oxaliplatin and retinoic acid, hybridized with core–shell nanoparticles, and magnetic field exposure	10 μg/mL

a Two samples of each group were analyzed.

After treatment, cells were detached with trypsin/EDTA, centrifuged, and resuspended in annexin V binding buffer. Cells were stained with annexin V-FITC and propidium iodide (PI) per manufacturer instructions (Invitrogen) and incubated for 20 min at room temperature in the dark. Samples were analyzed using a flow cytometer to detect fluorescence from FITC (530 nm, FL1) and PI (575 nm, FL3), and 10,000 events were recorded per sample.

#### 4.4.4 Uptake Assay

Uptake of nanoparticles by CT-26 cells was evaluated using fluorescence microscopy. Cells were seeded on 12 mm glass coverslips in 12-well plates (2 × 10^4^ cells/well). After adherence, cells were treated with CSNP, CGNP + OXA + CSNP, or CGNP + OXA + CSNP + RA for 24 or 48 h. Prior to treatment, nanoparticles were labeled with 10 μL of PI (100 μg/mL; Invitrogen) under constant agitation for 12 h. For plasma membrane labeling, Vybrant DiO (Molecular Probes) was used. After treatment, cells were fixed with 4% paraformaldehyde (Sigma-Aldrich), mounted with DAPI-containing medium (Abcam), and visualized using fluorescence microscopy.

### 4.5 In Vivo Studies

#### 4.5.1 Allotopic Colorectal Cancer Induction in BALB/c Mice

The colorectal cancer model was induced in BALB/c mice (6–10 weeks old) following the protocol described in a previous study.^20^ Mice were anesthetized with ketamine (100 mg/kg) and xylazine (10 mg/kg) and injected subcutaneously with 1 × 10^6^ CT-26 cells into the right flank. To minimize infection and discomfort, mice received enrofloxacin (10 mg/kg, SC, every 12 h for 3 days) and meloxicam (5 mg/kg, SC, every 24 h for 3 days). Tumor development was monitored, and treatments began when tumors reached ∼ 5 mm diameter (∼7 days postinduction). All procedures were approved by the UFRN Animal Ethics Committee (CEUA #323.059/2022).

#### 4.5.2 In Vivo Analysis of Antitumor Activity of Nanoparticles

BALB/c mice were randomly assigned to four groups (n = 4 per group) for treatment. The control group received a 0.9% NaCl solution at a dose of 5 mg/kg. The second group was treated with oxaliplatin (OXA) at a concentration of 7.5 mg/kg. The third group received a combination of cashew gum nanoparticles functionalized with oxaliplatin and core–shell nanoparticles (CGNP + OXA + CSNP) at the same dose. The fourth group received a similar formulation, but enriched with retinoic acid (RA), also at a final concentration of 7.5 mg/kg.

Treatment protocols were established based on the compounds and doses that exhibited the best responses in in vitro assays. The administration was performed intratumorally on days 7, 14, and 21 postinduction. On treatment days, groups 3 and 4, which received magnetic core–shell nanoparticles (CSNPs), were also exposed to a magnetic field. For this procedure, the mice were anesthetized via intraperitoneal injection with ketamine hydrochloride (100 mg/kg) and xylazine (10 mg/kg), followed by treatment administration. Immediately after injection, the animals were positioned with the tumor facing upward at the center of a copper solenoid and exposed to the magnetic field for 3 min. Groups 1 and 2 underwent treatment without magnetic field exposure.

Tumor growth was tracked through caliper measurements every 2 days after induction.^5^ After surgical removal, tumor volume was calculated using the formula: Tumor volume (mm^3^) = (width × length^2^) × 0.52.

#### 4.5.3 Histopathological Analysis

On the 23rd day postinduction, the mice were euthanized through intraperitoneal administration of ketamine hydrochloride (300 mg/kg) and xylazine (64 mg/kg), followed by cervical dislocation. After tumor excision, the samples were collected, fixed, processed, embedded in paraffin, and sliced into 5 μm sections for hematoxylin and eosin (H&E) staining to facilitate morphological assessment.

The evaluation was conducted in a double-blinded manner by two pathologists using optical microscopy (Nikon Eclipse 2000, equipped with a Nikon DS-Fi2 camera; Nikon Corporation, Tokyo, Japan). The scoring criteria encompassed four key parameters: the presence of anaplastic cells, the extent of inflammatory infiltration, the formation of blood vessels, and the degree of necrosis.^41^ At least three tumor sections per sample were analyzed at magnifications of 100× and 400×.

#### 4.5.4 Immunofluorescence

Immunofluorescence staining for superoxide dismutase-1 (SOD-1) and Hoechst 33342 was conducted following well established protocols.^42,43^ For both staining procedures, three sections measuring 3 μm were obtained from each tumor. Tissue samples underwent deparaffinization in xylene, followed by a graded ethanol rehydration sequence. Antigen retrieval was performed using a 10 mM sodium citrate buffer with 0.05% Tween 20. To minimize background fluorescence, slides were incubated for 20 min at room temperature in a 0.1% Sudan Black solution.

For SOD-1 labeling, a polyclonal anti-SOD-1 primary antibody (sc-8636, Santa Cruz, CA, USA) was diluted at 1:250 in 5% BSA and incubated at 4 °C for 24 h in a humidified chamber. Slides were then washed twice with PBS containing 0.2% Triton X-100 for 5 min and incubated with the Alexa Fluor 488 secondary antibody (1:500) for 1 h. Mounting was performed using Fluoroshield with DAPI mounting medium (ab104139, Abcam, Waltham/MA, USA).

For Hoechst 33342 staining, a 10 mg/mL stock solution was diluted at 1:100 in distilled water and applied to each section while protected from light. Incubation lasted 30 min at room temperature under constant agitation. Subsequently, slides were washed three times with PBS and mounted using Dako mounting medium (S3023, Dako, Carpinteria/CA, USA).

Images from both staining procedures were captured using a Carl Zeiss Laser Scanning Microscope (LSM 710, Carl Zeiss, Jena, Germany). Positive and negative controls were included as internal quality assessments. For group comparisons, the mean fluorescence intensity was measured using Zeiss ZEN lite software.

#### 4.5.5 Immunohistochemistry

Immunohistochemistry analysis of tumor tissue was conducted following a similar protocol used in our previous works.^35^ In summary, paraffin-embedded tumor samples underwent deparaffinization, rehydration, and antigen retrieval. The tissue was then incubated overnight at 4 °C with the primary Anti-Cox-2 antibody (Proteintech, Cat# 12375–1-AP, RRID:AB_2085127, 1:400), followed by a secondary antibody conjugated to horseradish peroxidase (HRP) (Boster Biological Technology, Cat# BA1054, RRID: AB_2734136, 1:50).

For visualization, diaminobenzidine (DAB; EasyPath, Cat# EP-12–20542) was applied, followed by counterstaining with hematoxylin. Tissue analysis was performed using a Nikon E200 LED light microscope (Minato) equipped with a Moticam digital camera.

Immunolabeling intensity was evaluated semiquantitatively by two pathologists under double-blind conditions. Staining intensity was classified into four levels: 1 = no staining, 2 = weak staining, 3 = moderate staining, and 4 = strong staining.^38^ Each histological section was divided into four quadrants, and the final score represented the average staining intensity across all quadrants.

### 4.6 Statistical Analysis

Data are presented as mean ± standard deviation (SD). Statistical analyses were performed using GraphPad Prism version 9.0 (GraphPad Software, San Diego, CA, USA). Normality was assessed using Anderson-Darling, D’Agostino-Pearson, and Shapiro-Wilk tests. For data sets with normal distribution, comparisons among multiple groups were performed using two-way ANOVA followed by Dunnett’s multiple comparisons test, while Bonferroni’s post hoc test was applied for intragroup comparisons across different time points, including those related to cell viability, uptake, and flow cytometry assays. For data sets that did not follow a normal distribution—including histopathological scores, immunohistochemistry (COX-2), immunofluorescence analyses (SOD-1 and Hoechst 33342), and tumor growth data—the Kruskal–Wallis test followed by Dunn’s multiple comparisons test was applied. A p-value of <0.05 was considered statistically significant for all analyses.

## 5 Conclusion

This study demonstrated that a multifunctional nanosystem composed of cashew gum nanoparticles coloaded with oxaliplatin and retinoic acid, combined with magnetic core–shell nanoparticles, enhances cytotoxicity and promotes apoptosis in colorectal cancer models. Under magnetic field stimulation, the nanosystem showed increased cellular uptake, significant induction of late apoptosis, and tumor tissue necrosis, while also modulating oxidative stress and COX-2 expression. Although tumor volume reduction was not observed, the histopathological and molecular outcomes strongly support the therapeutic relevance of this strategy. These findings highlight the potential of integrating magnetic stimulation, drug delivery, and biochemical modulation into a single platform for colorectal cancer treatment. Future work should focus on comprehensive biodistribution studies, pharmacokinetics, tumor penetration analysis, and the development of combinatorial approaches with immunotherapies to further advance the clinical translation of this nanoplatform.

## Supplementary Material



## Data Availability

The data underlying this study are available in the published article and its Supporting Information.

## Notes

Flow cytometry dot plots showing apoptosis profiles of CT-26 colorectal cancer cells after 48 h of treatment (Figure S1); flow cytometry dot plots showing apoptosis profiles of CT-26 colorectal cancer cells after 72 h of treatment (Figure S2); photographs of the experimental setup for magnetic hyperthermia assays, including equipment and configurations for *in vitro* and *in vivo* experiments (Figure S3) (PDF)

## Abbreviations

CRC: Colorectal cancer
CG: Cashew gum
CGNP: Cashew gum nanoparticles
CSNP: Core–shell nanoparticles
OXA: Oxaliplatin
RA: Retinoic acid
ATRA: All-trans-retinoic acid
EMT: Epithelial-mesenchymal transition
RAR: Retinoic acid receptor
RXR: Retinoid X receptor
PLA: Poly­(lactic acid)
DLS: Dynamic light scattering
PDI: Polydispersity index
SEM: Scanning electron microscopy
TEM: Transmission electron microscopy
DMEM: Dulbecco’s modified Eagle medium
PI: Propidium iodide
DAPI: 4′,6-Diamidino-2-phenylindole
MF: Magnetic field
COX-2: Cyclooxygenase-2
SOD-1: Superoxide dismutase-1
PBS: Phosphate-buffered saline
FBS: Fetal bovine serum
DMSO: Dimethyl sulfoxide
TEA: Triethylamine
PVP: Polyvinylpyrrolidone
AuNP: Gold nanoparticle
Au: Gold
Fe_3_O_4_: Magnetite
UV: Ultraviolet
LC: Inductance-capacitance
HRP: Horseradish peroxidase
DAB: Diaminobenzidine
TPGS: D-α-Tocopheryl polyethylene glycol 1000 succinate
ATCC: American Type Culture Collection
CEUA: Comissão de Ética no Uso de Animais.
