# Supplementary material for: Hybrid System of Magnetic Nanoparticles and Cashew-Gum Nanoparticles Induce Apoptosis and Tumor Necrosis in Colorectal Cancer
Source: ACS Omega. 2025 Dec 5;10(49):60205–19. doi: 10.1021/acsomega.5c06166 (PMC12713475; doi:10.1021/acsomega.5c06166)
Supplement: Supplementary file 1 [file ao5c06166_si_001.pdf]

Supporting Information files

Paper title: Hybrid System of Magnetic Nanoparticles and Cashew-Gum Nanoparticles Induces Apoptosis and Tumor Necrosis in Colorectal Cancer

Authors: Vinícius B. Garcia[1], Cainã O. G. da Silva[2], Luiz H. S. Gasparotto[3], Carlos A. M. Iglesias[4,8], Isadora L. G. da Silva [1,5], Emily L. Oliveira[1], Raelle F. Gomes[6], Regina C. M. de Paula[6], Rosemayre S. Freire[7], Felipe Bohn[8], Raimundo F. de Araújo Júnior[1,5*].

1. Inflammation and Cancer Research Laboratory, Department of Morphology, Federal University of Rio Grande do Norte (UFRN), 59078-970, Natal, RN, Brazil

2. Postgraduate Program in Structural and Functional Biology, Department of Morphology, Federal University of Rio Grande do Norte (UFRN), 59.072-970, Natal, RN, Brazil

3. Institute of Chemistry, Federal University of Mato Grosso (UFMT), 78060-900, Cuiabá MT, Brazil

4. Department of Physics, State University of Rio Grande do Norte, 59610-090, Mossoró, RN, Brazil

5. Postgraduate Program in Health Sciences, Federal University of Rio Grande do Norte (UFRN), 59078-970, Natal, RN, Brazil

6. Department of Organic and Inorganic Chemistry, Federal University of Ceará (UFC), 60455-760, Fortaleza, CE, Brazil

7. Department of Physics, Federal University of Ceará (UFC), 60455-760, Fortaleza, Brazil.

8. Department of Physics, Federal University of Rio Grande do Norte, 59078-900, Natal, RN, Brazil

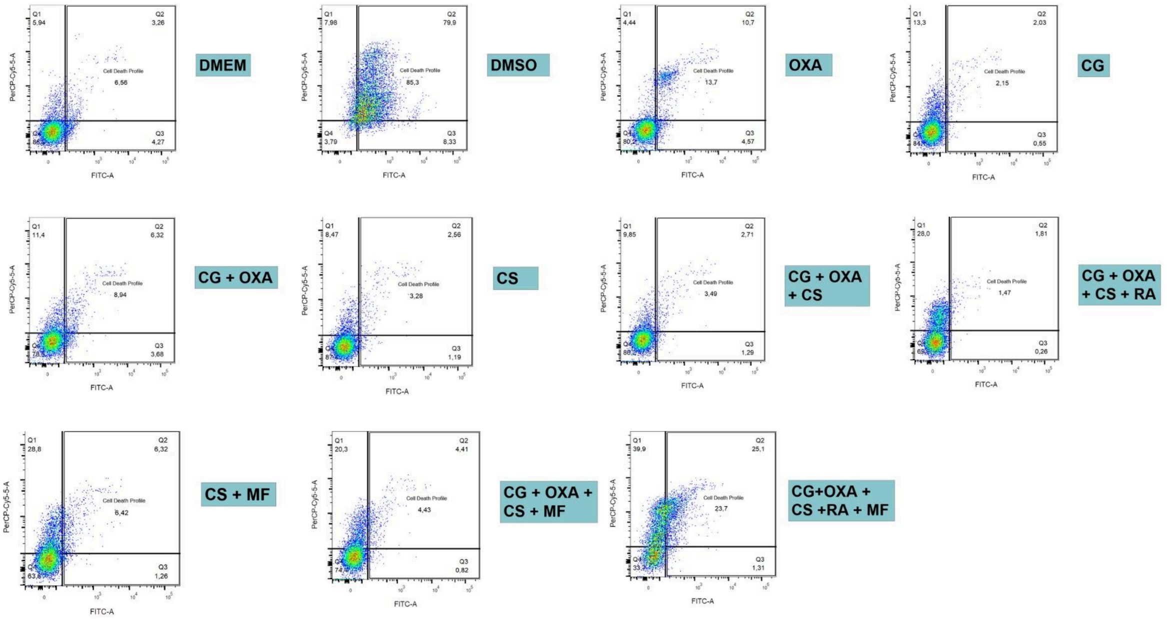

Figure S1. Flow cytometry dot plots showing the apoptosis profile of CT-26 colorectal cancer cells after 48 hours of treatment. Cells were stained with Annexin V-FITC and propidium iodide (PI) to distinguish viable cells (Annexin V$^-$/PI$^-$, lower left quadrant), early apoptotic cells (Annexin V$^+$/PI$^-$, lower right quadrant), late apoptotic cells (Annexin V$^+$/PI$^+$, upper right quadrant), and necrotic cells (Annexin V$^-$/PI$^+$, upper left quadrant). Experimental groups include: DMEM (negative control), DMSO (toxicity control), OXA (Oxaliplatin), CG (Cashew Gum nanoparticles), CG+OXA (Cashew Gum nanoparticles loaded with Oxaliplatin), CS (Core–Shell magnetic nanoparticles), CG+OXA+CS (hybrid nanoparticles without magnetic field), CG+OXA+CS+RA (hybrid nanoparticles functionalized with Retinoic Acid), CS+MF (Core–Shell nanoparticles under magnetic field stimulation), CG+OXA+CS+MF (hybrid nanoparticles under magnetic field stimulation), and CG+OXA+CS+RA+MF (fully functionalized hybrid nanoparticles under magnetic field stimulation). The highest proportion of late apoptotic cells was observed in the CG+OXA+CS+RA+MF group, followed by CG+OXA+CS+MF, indicating the synergistic pro-apoptotic effect of magnetic field stimulation combined with retinoic acid.

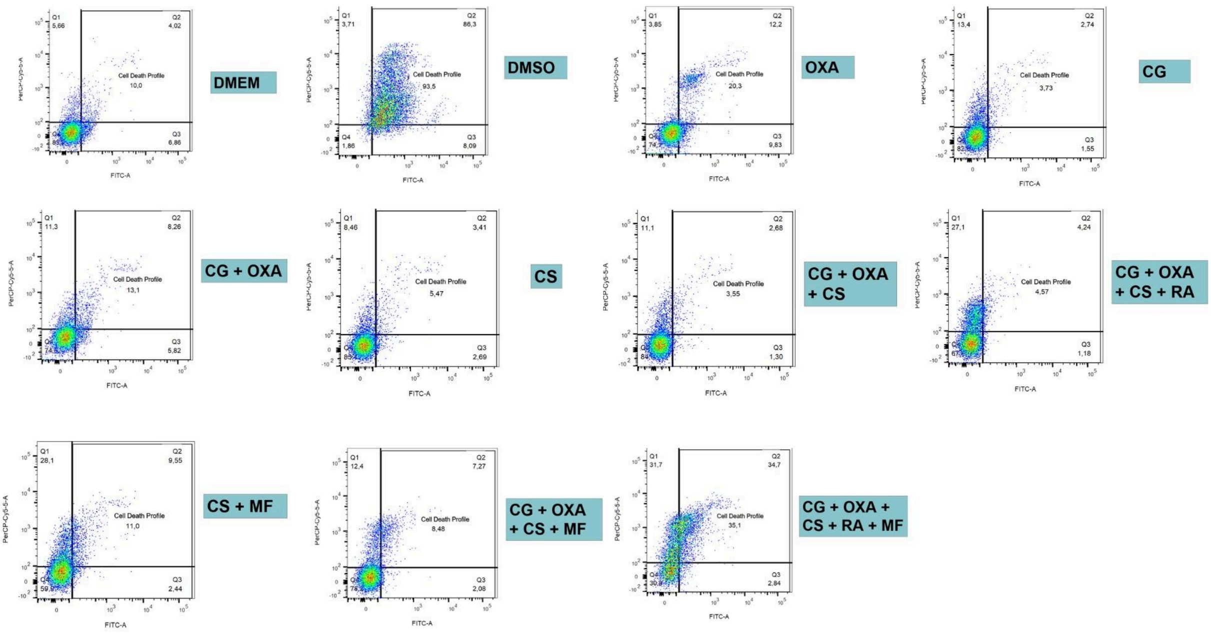

Figure S2. Flow cytometry dot plots showing the apoptosis profile of CT-26 colorectal cancer cells after 72 hours of treatment. Cells were stained with Annexin V-FITC and propidium iodide (PI) to distinguish viable cells (Annexin V^-/PI^-, lower left quadrant), early apoptotic cells (Annexin V^+/PI^-, lower right quadrant), late apoptotic cells (Annexin V^+/PI^+, upper right quadrant), and necrotic cells (Annexin V^-/PI^+, upper left quadrant). Experimental groups include: DMEM (negative control), DMSO (toxicity control), OXA (Oxaliplatin), CG (Cashew Gum nanoparticles), CG+OXA (Cashew Gum nanoparticles loaded with Oxaliplatin), CS (Core–Shell magnetic nanoparticles), CG+OXA+CS (hybrid nanoparticles without magnetic field), CG+OXA+CS+RA (hybrid nanoparticles functionalized with Retinoic Acid), CS+MF (Core–Shell nanoparticles under magnetic field stimulation), CG+OXA+CS+MF (hybrid nanoparticles under magnetic field stimulation), and CG+OXA+CS+RA+MF (fully functionalized hybrid nanoparticles under magnetic field stimulation). The CG+OXA+CS+RA+MF group displayed the highest proportion of late apoptotic cells, followed by CG+OXA+CS+MF, confirming that the combination of magnetic field stimulation and retinoic acid enhances the pro-apoptotic effect of the nanosystem. This pattern is consistent with the data observed at 48 hours and further supports the role of this combined therapeutic approach in promoting apoptosis in colorectal cancer cells.

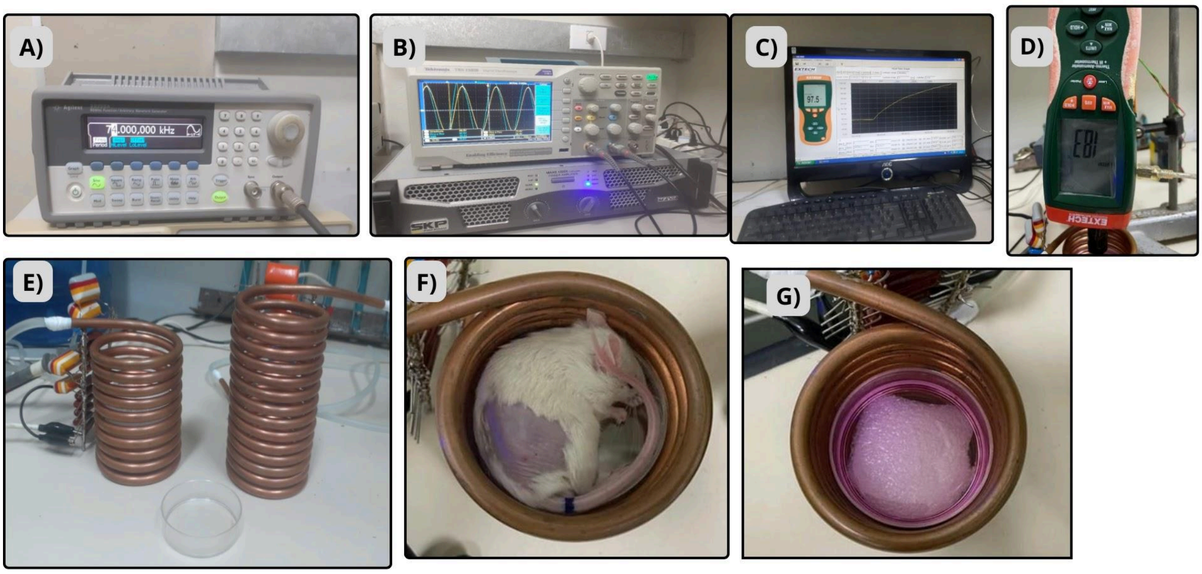

Figure S3. Experimental setup for magnetic hyperthermia assays. (A) Agilent 33250A waveform generator; (B) Mimipa MO-1222 oscilloscope connected to the SKP 1220X power amplifier; (C) Computer running Teledyne FLIR Extech HF300 2.3 software for signal control and monitoring; (D) Teledyne FLIR Extech HD300 infrared thermometer; (E) Custom-made copper solenoid coil designed for both *in vitro* and *in vivo* applications; (F) Solenoid containing a sedated mouse positioned on a 60 × 15 mm Petri dish for *in vivo* hyperthermia experiments; (G) Solenoid containing CT-26 cells (3 × 10^6 cells) cultured in a 60 × 15 mm Petri dish for *in vitro* hyperthermia assays. Although the infrared thermometer (D) is part of the system, it was not employed in the experiments because it lacked sufficient sensitivity to detect the temperature variations produced by the low concentrations of CSNP used in the in vitro (10 µg/mL) and in vivo (7.5 mg/kg) assays.
